# Brugada Pattern Phenocopy Induced by Diabetic Ketoacidosis

**DOI:** 10.7759/cureus.15066

**Published:** 2021-05-16

**Authors:** Eric Landa, Sasha Sharifi, Joseph Abraham, Erika Vigandt, Ethan Munzinger

**Affiliations:** 1 Internal Medicine, Unity Health, Searcy, USA; 2 Internal Medicine, Ross University School of Medicine, Bridgetown, BRB; 3 Interventional Cardiology, Unity Health, Searcy, USA

**Keywords:** brugada syndrome, phenocopy, dka, brugada pattern, brugada phenocopy

## Abstract

Brugada syndrome is a congenital cardiac channelopathy characterized by ST‐segment elevation (≥2 mm) and subsequent inverted T wave in a minimum of two right precordial leads (Brugada type 1 ECG [electrocardiogram] pattern) on ECG. Brugada syndrome is estimated to be responsible for 4%-12% of all sudden cardiac deaths and up to 20% in patients with structurally normal hearts. Development of a temporary Brugada pattern, known as Brugada phenocopy, has been observed in individuals presenting with reversible underlying conditions such as hyperkalemia, hyponatremia, acidosis, ischemia, and pulmonary embolism, among others. Herein we present a case of Brugada phenocopy seen in a patient in diabetic ketoacidosis, which resolved after the electrolyte abnormalities were corrected.

## Introduction

Affecting five out of 10,000 people worldwide, Brugada syndrome was first observed in 1986 in a young boy suffering from episodes of syncope and was later characterized in 1996 by Yan and Antzelevitch by highlighting the importance of the ST-segment elevation in V1-V3 along with an apparent right bundle branch block [1,2]. Brugada phenocopy, on the other hand, is an identical entity that demonstrates the same ECG pattern but is caused by various different factors. The Brugada pattern seen in these individuals always resolves once the underlying cause is fixed. Here we present such a case.

## Case presentation

**Figure 1:**
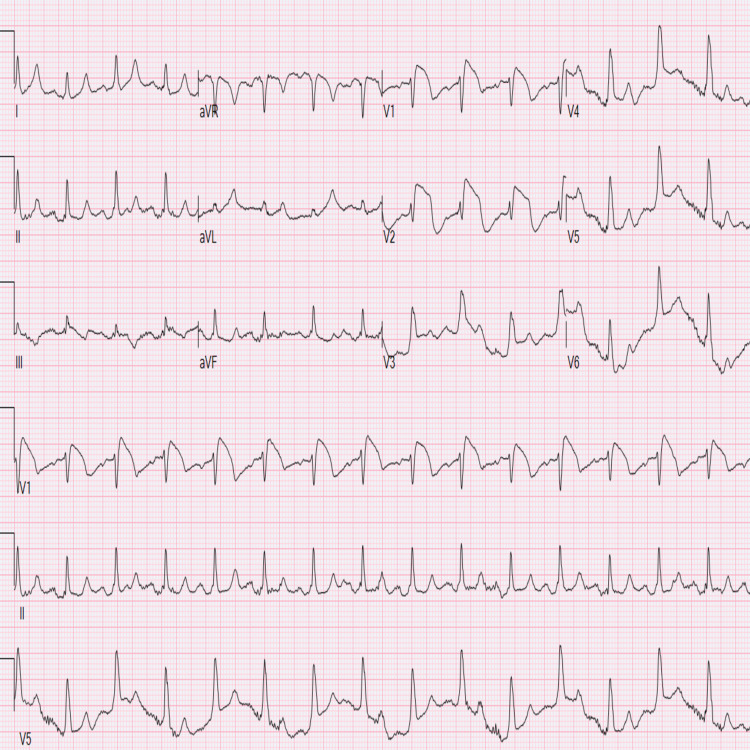
Electrocardiogram showing Brugada type I pattern in V1 and V2 leads

A 48-year-old Caucasian male with a past medical history of type I diabetes presented to the Emergency Department complaining of diffuse pain and fatigue. He began feeling tired three days prior to presentation with associated nausea and vomiting but denied any fever, chills, or chest pain. The patient stated that he had not been on an insulin pump at home and had only been using fast-acting insulin. This was his fifth presentation to the hospital for diabetic ketoacidosis (DKA). On arrival, his vital signs were as follows: temperature of 99.1°F, blood pressure of 129/68, pulse rate of 90 bpm, respiratory rate of 41 breathes per minute, and O_2_ saturation of 96% on room air. His labs were significant for a blood glucose of 952 mg/dL, sodium of 122 mmol/L, potassium of 7.6 mmol/L, bicarbonate of <5 mmol/L, anion gap of 34 mmol/L, beta-hydroxybutyrate of 11.84 mmol/L, hemoglobin A1C of 10.4%, white blood cell count of 38.6 thousand/uL, and pH of 6.94. Initial troponin was 0.04 ng/mL, which is clinically negative for a myocardial ischemia. Physical examination was significant for Kussmaul breathing and fruity breath. Electrocardiogram (ECG), as shown in Figure 1, showed ST-segment elevation in leads I-III, and the cath lab was activated. The cardiologist had some doubts about the ECG changes; therefore, he performed an echocardiogram, which showed no regional wall abnormalities and adequate left ventricular function. The patient went on to receive normal saline at a rate of 250 mL/hour; he was put on an insulin drip at 9 U/hour and brought to the intensive care unit.

**Figure 2:**
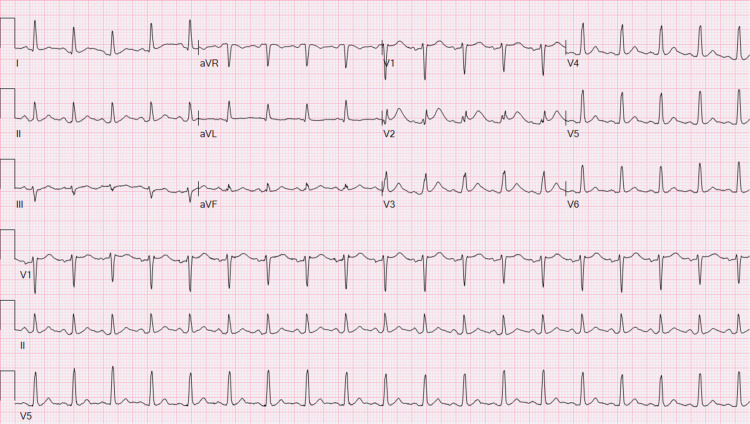
Electrocardiogram showing the resolution of Brugada type I pattern

As the patient continued to receive fluids along with the appropriate insulin regimen for his DKA, his anion gap closed at 10 mmol/L, beta-hydroxybutyrate became normal at 0.21 mmol/L, bicarbonate came back up to 21 mmol/L, potassium normalized to 4.6 mmol/L, and acidosis resolved. Interestingly, his new ECG no longer demonstrated a Brugada type I pattern (Figure 2).

## Discussion

Brugada syndrome is a rare and often fatal autosomal dominant genetic disorder. In 20% of cases, it is caused by a mutation in the alpha subunit of the cardiac sodium channel gene *SCN5A* [3]. Brugada syndrome has two forms, a congenital form and an acquired one. In addition to sodium channel blockers, a febrile state, alpha-agonists, beta-blockers, tricyclic antidepressants, vagotonic agents, and cocaine toxicity have been shown to uncover Brugada syndrome in those who have it [4-5]. There are three ECG repolarization patterns, as seen in Table 1, that are recognized in Brugada syndrome. Only type I is used as a criterion for diagnosis [6].

**Table 1:** Criteria for the different patterns seen in Brugada syndrome

	Type I	Type II	Type III
J wave amplitude	≥2 mm	≥2 mm	≥2 mm
T wave	Negative	Positive or biphasis	Positive
ST-T configuration	Coved type	Saddleback	Saddleback
ST segment (terminal portion)	Gradually descending	Elevated ≥1 mm	Elevated ≤1 mm

Many individuals with no history of syncopal episodes or a family history of sudden cardiac death have developed what is known as Brugada phenocopy. It is the appearance of the Brugada pattern on ECG, which is often confused with a STEMI, which appears when there is a metabolic derangement causing electrolyte imbalances, most notably hyperkalemia but also seen in hyponatremia, pH imbalances such as acidosis, ischemia, hypothyroidism, and compression [7]. The only established therapy for Brugada syndrome is implantation of a cardioverter-defibrillator, which is not a suitable answer for those around the world where access to a facility capable of implanting one is limited [8]. An experimental study that investigated pharmacological treatment of Brugada syndrome showed that both quinidine and tedisamil were effective in normalizing the ST-elevation seen in Brugada syndrome [9]. However, long-term efficacy has not been proven in preventing sudden cardiac death [10]. Brugada syndrome is an understudied phenomenon and thus continues to be studied. Brugada phenocopy is a recently discovered phenomenon, which still leaves much to be learned from. Here we presented a case of Brugada phenocopy in order to bring awareness to providers worldwide of the importance of early recognition and treatment of the underlying causes of this condition.

## Conclusions

Even though it is not a common event, Brugada phenocopy is a manifestation that can mislead even experienced physicians into making a wrong diagnosis as it can easily be confused with an STEMI. Whether caused by electrolyte abnormalities or a state of acidosis as seen in DKA, the treatment to obtain resolution of the Brugada pattern seen on ECG is to treat the underlying cause. Once the metabolic derangement has been resolved, then resolution of the Brugada pattern will follow. We presented such a case in order to help educate physicians about this phenomenon and its presentation.
